# Influence of NADPH oxidase on inflammatory response in primary intestinal epithelial cells in patients with ulcerative colitis

**DOI:** 10.1186/1471-230X-13-159

**Published:** 2013-11-14

**Authors:** Rima Ramonaite, Jurgita Skieceviciene, Gediminas Kiudelis, Laimas Jonaitis, Algimantas Tamelis, Paulius Cizas, Vilmante Borutaite, Limas Kupcinskas

**Affiliations:** 1 Institute for Digestive Research, Academy of Medicine, Lithuanian University of Health Sciences, A. Mickeviciaus str. 9, LT-44307 Kaunas, Lithuania; 2 Department of Gastroenterology, Academy of Medicine, Lithuanian University of Health Sciences, A. Mickeviciaus str. 9, LT-44307 Kaunas, Lithuania; 3 Department of Surgery, Academy of Medicine, Lithuanian University of Health Science, A. Mickeviciaus str. 9, LT-44307 Kaunas, Lithuania; 4 Institute for Neurosciences, Academy of Medicine, Lithuanian University of Health Sciences, A. Mickeviciaus str. 9, LT-44307 Kaunas, Lithuania

**Keywords:** Cell-biology, Cytokines, Ulcerative colitis, NADPH oxidase

## Abstract

**Background:**

The aim of this study is to evaluate the role of NADPH oxidase in primary intestinal epithelial cells during the active phase of UC.

**Methods:**

The primary human colonic epithelial cells were isolated from 19 patients with mild to moderate inflammatory activity of UC and 14 controls using chelation method. The cells were cultivated under the effect of mediators. Viability of cells was assessed by fluorescent microscopy. Production of reactive oxygen species (ROS) by the cells was measured fluorimetrically using Amplex Red. Production of TNF-α cytokine by the colonic epithelial cells was analysed by ELISA.

**Results:**

The results of our study showed that unstimulated cells of UC patients had a decreased viability, increased ROS production, but similar TNF-α level when compared to the controls. Stimulation with LPS increased hydrogen peroxide and TNF-α level in the UC group. Treatment of colonic epithelial cells with NADPH oxidase inhibitor increased cell viability decreased the levels of ROS and TNF-α in the LPS-treated cells isolated from UC patients.

**Conclusions:**

Our study showed that bacterial endotoxins induced NADPH oxidase activation in the colonic epithelial cells. Moreover, we revealed that treatment with NADPH oxidase inhibitors had a protective effect against pro-inflammatory action of LPS in human colonic epithelium cells during inflammation.

## Background

Ulcerative colitis (UC) is a chronic inflammatory bowel disease (IBD) that affects intestinal mucosa. The pathogenic mechanisms of UC are complex and involve interaction between genetic, host immune system and environmental factors. One of the major factors in the onset of UC is inappropriate mucosal immune response towards the intestinal microbiota leading to mucosal tissue damage and chronic inflammation [1-3].

Increased production of reactive oxygen species (ROS) and oxidant-induced protein or lipid alterations have been implicated in tissue damage observed in chronic inflammatory disorders, such as IBD [4]. The key producers of the superoxide anions in the colon are non-phagocytic and phagocytic cells. The epithelial NADPH oxidase homologs (Nox1, Nox3, Nox4, Nox5, DUOX1, and DUOX2) generate a higher level of superoxide in the colon compared to phagocyte NADPH oxidase [5]. Previous studies have shown that epithelial NADPH oxidase mediating formation of ROS might be involved in host defence system and inflammatory responses at mucosal surfaces [6-8]. ROS production in the intestinal mucosal biopsies is increased during inflammation [9]. Moreover, genetic mutations in genes encoding components of the NADPH complex have been associated with IBD susceptibility. The variations have been found in genes responsible for localization of the NADPH oxidase complex (including p47phox and p67phox and RAC2) to the membrane [10,11].

Despite the profound consequences of either absent or excessive ROS generation in the intestinal tract, little is known about the molecular pathways controlling ROS production via NOX enzymes in the primary intestinal epithelial cells derived from UC patients. Therefore, in this study we aimed to evaluate the role of NADPH oxidase in primary intestinal epithelial cells during the active phase of UC.

## Methods

### Patients

The colonoscopic biopsies were obtained from 19 patients with UC (men n = 9 (medium age ± SD = 45.8 ± 20.1), women n = 10 (medium age ± SD = 42 ± 18)) and 14 control subjects (men n = 7 (medium age ± SD = 44 ± 19.5), women n = 7 (medium age (years ± SD) = 47 ± 15.3)). UC patients and control subjects were recruited in the Department of Gastroenterology, Hospital of Lithuanian University of Health Sciences. The diagnosis of UC was based on standard clinical, endoscopic, radiological, and histological criteria [12-14]. Patients with mild to moderate disease activity were included in the study (Mayo UC Endoscopic Score 1 to 2). Histologically, these patients had active chronic UC as well. The individuals did not use steroid or immunosuppressive therapy at least 3 months before the biopsies specimens have been obtained. Only five patients had used 5-aminosalicylate (5-ASA) preparations as maintenance therapy (≤ 1.5 g/d). None of the patients had received iron supplementation.

The control group consisted of patients with irritable bowel disease or functional constipation. Individuals were included if they had a normal colonoscopy and un-inflamed mucosa on histopathological examination. All patients had a routine colonoscopy performed as a part of their planned examination programme. In healthy subjects, eight to ten biopsies (5–10 mg wet weight each) were taken from transverse or descending colon. The same numbers of biopsies from patients with UC were collected from endoscopically inflamed colonic mucosa. Biopsies were immediately placed in a chilled Dulbecco’s modified Eagle medium containing 10 mM HEPES buffer and antibiotics (50 IU/ml penicillin, 50 mg/ml streptomycin and 0.5 mg/ml of gentamicin). Written informed consent was obtained from all study participants. The study has been approved by the Kaunas Regional Biomedical Research Ethics Committee (Protocol No. BE – 2–49).

### Isolation and cultivation of primary human colonic epithelial cells

All chemicals were obtained from Sigma-Aldrich (Steinheim*,* Germany) unless otherwise stated. The primary human colonic epithelial cells were isolated using chelation method according to Seidelin *et al.*[15]. The incubation time in the chelation buffer lasted for 40–45 min at room temperature that allowed the isolation of single epithelial cells. The isolated epithelial cells were suspended in DMEM with 15% fetal calf serum (FCS), 10 mM HEPES buffer and antibiotics. Approximately 10^7^ cells per well were cultured with mediators (20 μg/ml of lipopolysaccharide (LPS), 1 mM of apocynin, 20 μg/ml LPS + 1 mM apocynin, 200 units/ml of catalase) in 24-well plates coated with bovine dermal collagen for 24 hours at 37°C in an atmosphere of 5% CO_2_ and at 90% relative humidity. After 24 hours, supernatants were collected and stored at -20°C.

### Measurement of hydrogen peroxide production

NADPH oxidase generates superoxide, which is rapidly converted to hydrogen peroxide [16]. Hydrogen peroxide production in cells was measured fluorimetrically using 1 μM Amplex Red and 10 units/ml Horseradish peroxidase. Colonic epithelial cells (approximately 10^5^ cells/ml) were re-suspended in the phosphate-buffered saline (PBS) and incubated with either 5 units/ml of catalase (endogenous control), or 10 μg/ml of LPS, or 10 μM of diphenylene iodonium (DPI), or 10 μg/ml LPS + 10 μM DPI for 30 min at 37°C. Then the Amplex Red and peroxidase were added and the rate of hydrogen peroxide production was quantitated at 544 nm excitation and 590 nm emission by a microplate fluorometer (Fluoroskan Ascent, Thermo Fisher Scientific, Waltham, MA).

Apocynin (used as NADPH oxidase inhibitor during cell cultivation) interferes with detection of ROS in assay systems selective for hydrogen peroxide or hydroxyl radicals. This inhibitor acts as a radical scavenger and inhibits Amplex Red oxidation. Thus ROS are not measured accurately and cannot reflect the effect of apocynin on the NADPH oxidase activity [17]. Therefore, for the assessment of NADPH oxidase activity we applied another large-spectrum inhibitor DPI.

### Assessment of cell viability

The viability of human colonic epithelial cells in the cultures was assessed by propidium iodide (PI, 7 μM) and Hoechst 33342 (4 μg/ml) staining using a fluorescence microscope (OLYMPUS IX71S1F-3, Tokyo, Japan). PI-negative cells with weak Hoechst-staining were considered to be viable, whereas cells showing nuclear shrinkage or fragmentation and intensive Hoechst staining but still lacking PI staining were classified as chromatin condensed/fragmented (apoptotic). PI-positive cells were classified as necrotic. Human colonic epithelial cells were counted in at least 5 microscopic fields per well (three wells per treatment). Data are expressed as percentage of viable, necrotic or apoptotic cells of the total number of cells per field.

### Assessment of TNF -α concentration

Concentration of TNF-α was assessed in the supernatants of primary colonic epithelial cells cultures using commercially available two-site ELISA kit (Invitrogen, Carlsbad, CA). The lowest limit of sensitivity of test systems for TNF-α was 3 pg/ml. The optical densities at 450 nm and at a correction wavelength of 490 nm were measured on ELISA micro plate reader (MRX micro plate reader, Dynex Technologies, Denkendorf, Germany).

### Statistical analysis

Statistical analyses were done using the SPSS statistical package (version 16.0; Chicago, IL). Data in text and figures are presented as means ± standard error (SE). The results were analysed by one-way ANOVA. The least significant difference (LSD) test was used as a post hoc test. P-value of <0.05 was accepted as statistically significant.

## Results

### Assessment of viability of human colonic epithelial cells

**Figure 1:**
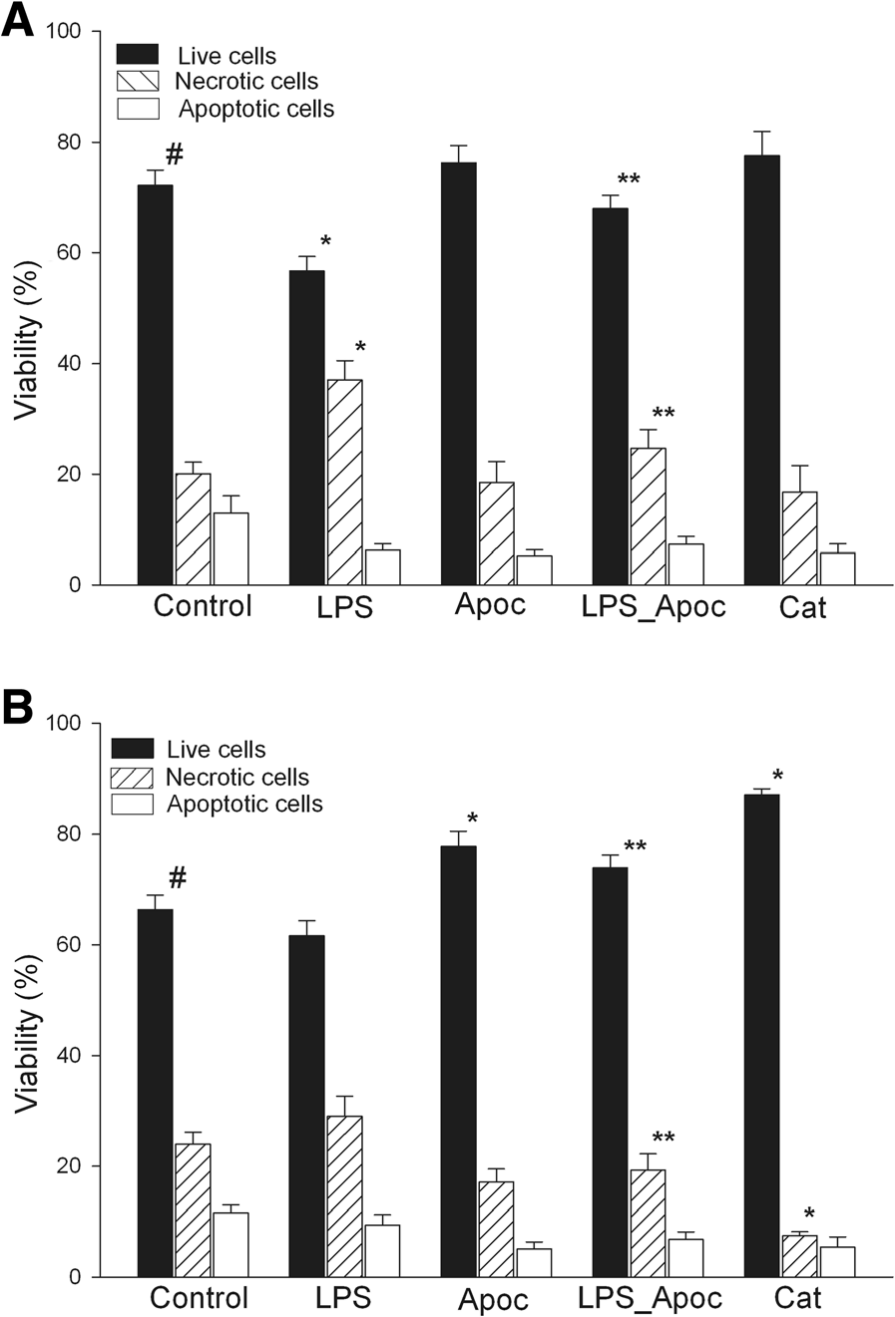
**Assessment of viability of human colonic epithelial cells.** Where indicated, cells were incubated with 20 μg/ml LPS, 1 mM apocynin (apoc), 20 μg/ml LPS + 1 mM apocynin, 200 units/ml catalase (Cat), and control without stimulation for 24 h. **A** - control group, **B** - UC group. ^*^Statistically significant difference between control and another subgroups in the control or UC groups. ^**^Statistically significant difference between LPS and LPS + Apocynin subgroups in the control or UC groups. ^#^Statistically significant difference between control and UC groups. Statistically significant, as p <0.05.

Firstly, we investigated whether there was any difference in survival of colonic epithelial cells isolated from UC patients and control individuals in cell culture. As shown in Figure 1, after 24 h in culture the vast majority of the control cells were viable and accounted for 72%, necrosis was observed in 18% and apoptosis in 10% of cells. The viability of cells isolated from UC patients and maintained in cell culture for 24 h was significantly lower than in control group (Figure 1A and B). Treatment of cells with NADPH oxidase inhibitor apocynin or with catalase (which selectively removes H_2_O_2_) increased the viability of the UC group cells reaching the survival level of untreated cells. In contrast, apocynin as well as catalase had no effect on cell survival in the control group.

Stimulation of colonic epithelial cells with LPS significantly reduced the number of live cells from 72% to 57% and increased necrotic cell death from 18% to 37% in the control group; whereas the viability of cells in UC group remained unchanged upon LPS treatment (Figure 1B). The cultivation of cells with LPS and apocynin increased the viability of the cells in the control and UC groups (to 76% and 78%, respectively) by decreasing the number of necrotic cells (Figure 1A and B). The percentage of apoptotic cells was 5–8% in all study groups.

### Assessment of hydrogen peroxide production in cells

**Figure 2:**
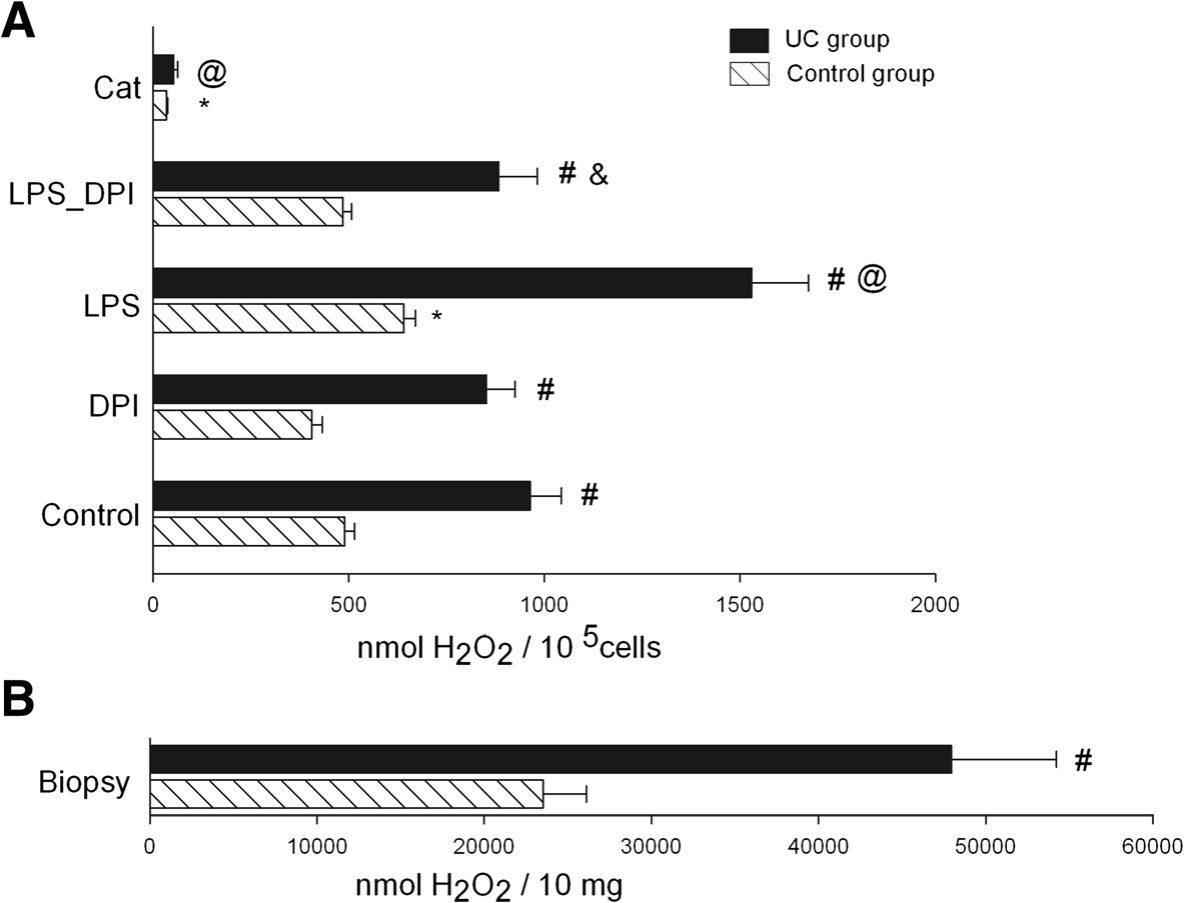
**Assessment of hydrogen peroxide production. A** - colonic epithelial cells were incubated for 30 min with 5 units/ml catalase (Cat), 10 μg/ml LPS, 10 μM DPI, 10 μg/ml LPS + 10 μM DPI or control without stimulation (where indicated). **B** - colonic biopsies (10 mg) of UC patients and control subjects were incubated without stimulation for 30 min. ^*^Statistically significant difference between control and another subgroups in the control group. ^@^Statistically significant difference between control and another subgroups in the UC group. & Statistically significant difference between LPS and LPS + DPI subgroups in the UC group. ^#^Statistically significant difference between control and UC groups. Statistically significant, as p <0.05.

Further, we analysed the ROS generation in primary colonic epithelial cells. As shown in Figure 2, the level of extracellular hydrogen peroxide production was approximately two times higher in epithelial cells of UC patients compared to the control group. Similar results were obtained when hydrogen peroxide production was measured directly in the colonic biopsies (Figure 2B). The treatment of colonic epithelial cells with catalase significantly decreased the level of extracellular hydrogen peroxide production in both experimental groups.

DPI slightly decreased the level of ROS production in both UC and control groups, however, the differences were not statistically significant. The treatment of cells with LPS significantly increased the production of hydrogen peroxide in cells of both study groups (Figure 2A). The addition of DPI to LPS-treated colonic epithelial cells significantly decreased (by 1.7 fold) the level of ROS production in the UC group. In control group, DPI also diminished hydrogen peroxide production in LPS-treated cells, though the effect of DPI *per se* was not statistically significant due to higher variability of results (Figure 2A).

### Assessment of TNF-α concentration in the colonic epithelial cells

**Figure 3:**
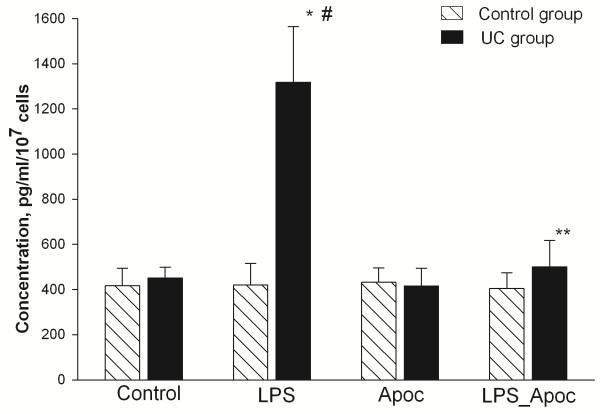
**Assessment of TNF-α production by human colonic epithelial cells.** Cells were incubated for 24 h with 20 μg/ml of LPS, 1 mM of apocynin (Apoc), 20 μg/ml LPS + 1 mM apocynin or control without stimulation (as indicated). After incubation cell media were collected and TNF-α concentration was measured as described in Methods. ^*^Statistically significant difference between control and LPS subgroups in the UC group. ^**^Statistically significant difference between LPS and LPS + apocynin subgroups in the UC group. ^#^Statistically significant difference between LPS subgroup in the UC and control groups. Statistically significant, as p <0.05.

In addition, we analysed the influence of NADPH oxidase on the production of pro-inflammatory cytokine TNF-α. As shown in Figure 3, the highest concentrations of TNF-α were determined in cells of UC patients after stimulation with LPS. The level of cytokine production was approximately three-fold higher in LPS treated UC cells when compared to UC cells without stimulation and control group cells treated with LPS. In the UC group, treatment of cells with LPS and NADPH oxidase inhibitor apocynin decreased the levels of TNF-α production approximately 2.5-fold as compared with the LPS treated colonic epithelial cells. The differences of TNF-α concentration between untreated and treated cells in the control group were insignificant.

## Discussion

Functional studies have indicated that increased activity of NADPH oxidase contributes to the development of colon inflammation [5,18]. Inhibition of this enzyme represents an attractive therapeutic target for the treatment of many diseases. Apocynin has been used as an inhibitor of the complex NADPH oxidase in many experimental models of inflammation involving phagocytic and non-phagocytic cells [19-21]. In this study, we examined the influence of superoxide generating NADPH oxidase in primary intestinal epithelial cells during the active phase of UC.

In the current study we showed that unstimulated cells of UC patients had a decreased viability, increased ROS production, and similar TNF-α level when compared to the control group. These findings are characteristic of UC as increased cell death and excessive ROS production are typical processes on-going during inflammation. Previous studies have shown that the level of TNF-α may correlate with the grade of inflammation in UC [22,23]. Similar levels of TNF-α observed in UC and control groups could be linked with absence of severe disease activity cases within our cohort of UC patients.

The results of our study showed that sensitisation of colonic epithelial cells with bacterial products were required for activation of NADPH oxidase. Cell viability in normal epithelial cells was dramatically decreased in the presence of LPS and assessment of extracellular hydrogen peroxide production showed increase in ROS production. This observation suggests that innate immune system may activate protective cascades against microbial invaders; whereas minor changes in TNF-α level may indicate suspended immune response towards microbial products. It is well known that ROS production is rapidly elevated during infection, serving to facilitate pathogen clearance as well as contributing to signalling cascades related to inflammation, cell proliferation, and immune responses [24]. In the UC group the response to microbial stimulation resulted in an increased production of oxidants and pro-inflammatory cytokine [24,25]. LPS-induced inflammatory responses are more intense and acute in UC patients because mechanisms responsible for bacterial recognition are unbalanced [25]. However, we cannot exclude the possibility that the presence of phagocytic cells might have affected ROS production and TNF-α concentration in the cell cultures from UC patients.

Animal models and *in vitro* cell culture studies have shown that apocynin possesses anti-inflammatory activity [19] and protects non-phagocytic cells from damage induced by bacterial products and can reduce damage in the colon tissues [26-28]. However, the impact of NADPH oxidase inhibitory components has not been previously shown in primary colon epithelial cell cultures. The impact of apocynin in our control group was minor; whereas in the UC group the inhibitor significantly increased cell viability and reduced TNF-α in the LPS-treated colonic epithelial cells. These findings indicate that NADPH oxidase inhibition has an anti-inflammatory effect in human colonic epithelium cells during inflammation. Similar findings have been observed in lung endothelial cells during inflammation. The suppression of NADPH oxidase activity by apocynin in the lung endothelial cells resulted in a significant reduction of all parameters of inflammation measured, including TNF-α level [29,30].

The results of this study confirmed that NADPH oxidase is directly involved in LPS-induced ROS generation in the primary colonic epithelial cells and revealed the inflammation reducing effect of NADPH oxidase inhibitors. Based on the findings observed in the studies performed in the cancer cell cultures [27,31], we suggest that a molecular mechanism for activation of NADPH oxidase in colonic epithelial cells may be associated with a toll-like receptor (TLR) pathway, where LPS strains potently stimulate ROS production by colon NADPH oxidase through a TLR4 [31-33]. Further studies should be designed to determine the mechanism of LPS/TLR4/TNF-α/colon NADPH oxidase signalling in colonic epithelial cells and its contribution to UC etiopathogenesis.

## Conclusion

In conclusion, we showed that bacterial endotoxins were required for NADPH oxidase activation in the colonic epithelial cells. Moreover, we revealed that treatment with NADPH oxidase inhibitors had a protective effect against pro-inflammatory action of LPS in human colonic epithelium cells during inflammation.

## Competing interests

The authors declare that they have no competing interests.

## Authors’ contributions

RR, JS – Performed experiments, analysed data, interpreted results of experiments, prepared figures, drafted manuscript. PC – Performed experiments, analysed data. GK, LVJ, AT – interpreted results of experiments, edited and revised manuscript. VB, LK – Conception and design of research, interpreted results of experiments, approved final version of manuscript. All authors read and approved the final manuscript.

## Pre-publication history

The pre-publication history for this paper can be accessed here:

http://www.biomedcentral.com/1471-230X/13/159/prepub
